# Use of Cepheid Xpert Carba-R® for rapid detection of carbapenemase-producing bacteria in critically ill, abdominal surgical patients: first report of an observational study

**DOI:** 10.1186/cc14188

**Published:** 2015-03-16

**Authors:** A Cortegiani, V Russotto, P Capuano, G Tricoli, DM Geraci, A Ghodousi, L Saporito, G Graziano, A Giarratano

**Affiliations:** 1 University of Palermo, Italy

## Introduction

Xpert Carba-R® (Cepheid®, USA) is a PCR-based assay for rapid (<1 hour) detection of bacteria carrying carbapenem-resistance genes (KPC, NDM, VIM, OXA-48, IMP-1). The aim of the study is to compare PCR with microbiological cultures in critically ill, abdominal surgical patients.

## Methods

We performed an observational study at University Hospital 'P. Giaccone' Palermo. We enrolled abdominal surgical patients admitted to the ICU with suspected abdominal sepsis or developing sepsis during the ICU stay. We obtained two rectal swab specimens and two drainage samples to perform PCR assay and classic culture tests. We used Cohen's *K *to test concordance of results. We considered concordant those results of positive detection of carbapenemase-producing bacteria by both methods (even if a polymicrobial growth was observed by cultures) or negative results by both methods. Concordance was studied for rectal swab and drainage specimens. Antibiotic susceptibility testing was performed through a semiquantitative method.

## Results

Eight complete samples sets were collected from seven patients. Seven rectal swab specimens were negative for both PCR and cultures. In one patient a positive culture from carbapenem-resistant *P. aeruginosa *was detected from the rectal swab resulting negative to PCR. In one patient a positive culture from carbapenem-resistant *A. baumanii *was detected by drainage culture resulting negative to PCR. In two cases a positive result was observed from both PCR and cultures of rectal swab and drainage specimens. Vim and KPC genes were detected in one case and *A. baumanii *and *K. pneumoniae *with carbapenem resistance were isolated from cultures. A KPC gene was detected by PCR in the other case, and *K. pneumoniae *with carbapenem resistance was isolated from cultures. In all other cases a negative result was observed by both PCR and cultures. Cohen's *K *of 0.71 (95% CI = 0.21 to 1) was observed for rectal swab and drainage specimens.

## Conclusion

We need more data to evaluate the performance of PCR for rapid detection of carbapenemase-producing bacteria from rectal swabs and drainage of critically ill surgical patients even though its concordance with cultures seems to be good.

